# Double Muscling in Cattle: Genes, Husbandry, Carcasses and Meat

**DOI:** 10.3390/ani2030472

**Published:** 2012-09-20

**Authors:** Leo O. Fiems

**Affiliations:** Animal Sciences Unit, The Institute for Agricultural and Fisheries Research (ILVO), Scheldeweg 68, B-9090 Melle, Belgium; E-Mail: leo.fiems@ilvo.vlaanderen.be; Tel.: +32-9-2722610; Fax: +32-9-2722601

**Keywords:** double muscling, myostatin, carcass quality, organ size, locomotion, reproduction, stress susceptibility, meat quality

## Abstract

**Simple Summary:**

Selection for an increased meatiness in beef cattle has resulted in double-muscled (DM) animals, owing to the inactivation of the myostatin gene. These animals are characterized by an excellent conformation and an extremely high carcass yield, coinciding with a reduced organ mass. As a consequence, voluntary feed intake is reduced, but feed efficiency is considerably improved, although maintenance requirements are not clearly reduced. DM animals are more susceptible to respiratory disease, stress and dystocia, requiring extra attention for accommodation and welfare. Carcasses of DM animals are very lean, and intramuscular fat content is low. The fatty acid profile is different when compared with non-DM animals, containing less saturated fatty acids. Collagen content of the meat is lower, so that meat from double-muscled animals is mostly more tender. However, meat tenderness, color and juiciness are not always improved. A different metabolism as a consequence of faster glycolytic myofibers can be partly responsible for this phenomenon. DM animals are interesting for the producer and butcher, and beneficial for the consumer, if an appropriate nutrition and accommodation, and adequate slaughter conditions are taken into account.

**Abstract:**

Molecular biology has enabled the identification of the mechanisms whereby inactive myostatin increases skeletal muscle growth in double-muscled (DM) animals. Myostatin is a secreted growth differentiation factor belonging to the transforming growth factor-β superfamily. Mutations make the myostatin gene inactive, resulting in muscle hypertrophy. The relationship between the different characteristics of DM cattle are defined with possible consequences for livestock husbandry. The extremely high carcass yield of DM animals coincides with a reduction in the size of most vital organs. As a consequence, DM animals may be more susceptible to respiratory disease, urolithiasis, lameness, nutritional stress, heat stress and dystocia, resulting in a lower robustness. Their feed intake capacity is reduced, necessitating a diet with a greater nutrient density. The modified myofiber type is responsible for a lower capillary density, and it induces a more glycolytic metabolism. There are associated changes for the living animal and post-mortem metabolism alterations, requiring appropriate slaughter conditions to maintain a high meat quality. Intramuscular fat content is low, and it is characterized by more unsaturated fatty acids, providing healthier meat for the consumer. It may not always be easy to find a balance between the different disciplines underlying the livestock husbandry of DM animals to realize a good performance and health and meat quality.

## 1. Introduction

There is a persisting trend to improve carcass quality in specialized beef breeds [1,2,3]. A higher meat yield and more lean meat are desirable for the meat industry [4]. A smaller fifth quarter is more interesting for both the producer and the butcher, resulting in more edible meat cuts. It is also beneficial for the consumer, as leaner meat may be favorable with regard to coronary and heart diseases [5]. 

Double-muscled (DM) animals produce very lean meat. They can be classified as an extreme type of late maturing animals [6]. They are often defined as animals with muscle hypertrophy. However, there is also some muscle hyperplasia besides muscle hypertrophy. Several beef breeds are characterized by the double-muscling phenomenon: A lot of research was done in the 1970s with Charolais DM animals [7]. Other typical breeds for the presence of DM animals are Piedmontese [8] and Belgian Blue breeds [9]. Animals with the double-muscled character are also reported for Asturiana de los Valles [10] and Marchigiana [11] breeds.

According to Darwin [12] in his book “The Origin of Species”, “it cannot be supposed that all the breeds were suddenly produced as perfect and as useful as we now see them; indeed, in many cases, we know that this has not been their history. The key is man's power of accumulative selection; nature gives successive variations: man adds them up in certain directions useful to him. In this sense he may be said to have made for himself useful breeds”. The exploitation of the double-muscling phenomenon is clearly the effort of man to improve the meatiness of beef cattle. The Belgian Blue breed is a typical example of the exploitation of the myostatin gene. A review of the characteristics of DM animals is extant [13], but this paper aims to relate different characteristics of double muscling to feed intake, health, reproduction, histology and metabolism, and carcass and meat quality, and defines the consequences for livestock husbandry. In referring to DM animals it is inferred that they are homozygote for myostatin; if heterozygote animals are involved, it is explicitly mentioned.

## 2. Origin of DM Animals: The Unraveling of the Myostatin Gene and Its Expression

Double muscling in cattle has been reported for more than a century [14]. However, due to the difficulties at parturition and the use of caesarean operations to assist calf birth, double muscling was only fully exploited after the Second World War, mainly because of the availability of anesthesia, antibiotics and new surgical methods.

Charlier *et al*. [15] have mapped a locus, referred to as mh (muscular hypertrophy), on bovine Chromosome 2, confirming the validity in the Belgian Blue population of the monogenic model involving an autosomal mh locus. The MSTN (myostatin) gene has been identified as the cause of the double-muscled phenotype in cattle [16]. An 11 base-pare deletion of the protein is responsible for muscular hypertrophy in Belgian Blue DM (BBDM) cattle. This naturally occurring mutation results in an inactivation of the MSTN gene and provokes a massive increase in the skeletal muscle mass. McPherron and Lee [17] reported the similarity in phenotypes of DM cattle and mice, carrying a targeted deletion of the MSTN gene (MSTN null mice, MSTN knock-out mice), suggesting that MSTN performs the same biological function in these two species. This similarity in biological processes between cattle and mice has been confirmed [18]. This statement means that MSTN may be a useful target for genetic manipulation in other farm animals. MSTN is a member of the transforming growth factor-β superfamily that encompasses a large group of secreted growth and differentiation factors playing important roles in regulating development and tissue homeostasis [17]. Gerrard *et al*. [19] showed that the presence of a co-twinned fetus with a lower genetic potential for muscle development reduced the capacity of heavily muscled fetuses to develop muscle mass, and they concluded that blood-borne factors regulate muscle hypertrophy in fetal cattle. MSTN expression is detected in the myotome early in myogenesis through to adult skeletal muscle, where it may control fiber number and size during embryonic, fetal, and postnatal myogenesis [9]. The existence of circulating forms of MSTN was reported [20,21]. MSTN is secreted under a latent form due to its association with inhibitory proteins [22,23]. Members of the bone morphogenetic protein-1 (BMP)/tolloid family of metalloproteinases can activate latent MSTN [24]. MSTN signals through a transforming growth factor-β/activin/nodal-like pathway and binds to activin IIB receptors, and, to a lesser extent, to activin IIA receptors, and partners with a Type I receptor, either activin receptor-like kinase 4 (ALK4 or ActRIB) or ALK5, which in turn initiates the intracellular signaling cascade by phosphorylation of the receptor-regulated proteins SMAD2 and SMAD3 [20,25]. MSTN potently antagonizes BMP7 but not BMP2 by competing for BMP7 binding to the ActRIIB type II receptor [25]. MSTN also auto-regulates its expression by feedback loop through a SMAD7 dependent mechanism [26]. However, muscle hypertrophy observed in constitutive MSTN knock-out mice is primarily due to myofiber hypertrophy, while that observed in MSTN-null cattle results primarily from hyperplasia [27]. The investigation of the protein alterations in the *semitendinosus* muscle in response to an 11-bp deletion in the MSTN gene showed that thirteen proteins are significantly altered between BBDM and non-DM animal muscles. Eight proteins are related to the contractile apparatus, two proteins are involved in metabolic pathways, and three proteins are significantly altered including sarcosin, sarcoplasmic reticulum 53 kDa glycoprotein and 20 kDa heat shock protein [27].

McPherron and Lee [17] also sequenced the MSTN gene in the Piedmontese breed. The similar map positions of the MSTN gene and the muscular hypertrophy locus and the identification of relatively severe mutations in the MSTN gene of two different DM cattle breeds suggest that these mutations are responsible for the DM phenotype. Mutations in Belgian Blue and Piedmontese DM cattle are different [9]. The entire MSTN coding sequence in ten European cattle breeds was determined [28]. The observation that in at least eight of the ten studied breeds double muscling involves five independent MSTN mutations indicates that the number of genes susceptible to affect muscular development in a comparable way is likely to be limited in cattle. 

The role of MSTN in the regulation of cell growth and cell death occurs in concert with insulin-like growth factor (IGF)-I [29]. It has been shown that the maximum expression of muscle IGF-II is delayed in DM animals during fetal development [30]. A differential regulation of the IGF-II gene transcription between DM and normal fetuses was also reported [31]. Insulin-like growth factor binding proteins (IGFBP-3 and IGFBP-5) affect processes downstream the receptor-mediated SMAD phosphorylation, enabling MSTN to suppress proliferation of porcine embryonic myogenic cells [32]. The role of IGF and IGFBP was also confirmed in mice [33]. MSTN null mice have higher circulating IGF-I levels than wild-type mice, whereas IGFBP-1 and IGFBP-2 levels were lower. Furthermore, it is assumed that the relative IGF-binding capacity was potentially lower in MSTN null mice. Muscle regulatory factors (MRF4, Myf5, MyoD and myogenin) play a key role in muscle development and maturation [34,35]. A distinct pivotal function in muscle development is attributed to each of these muscle regulatory factors. The expression of MyoD in fetal bovine muscles is up-regulated by the loss of functional MSTN [36,37,38]. Higher levels of MyoD expression throughout primary and secondary fiber formation were found in BBDM fetuses compared to non-DM fetuses [37,38]. Higher expressions of MRF4, Myf5 and MyoD were found in DM than in non-DM Japanese Shorthorn cattle, but the effect was different between muscles [39]. Furthermore, the effect of MSTN on MRF4 expression may be more specific for fast type muscle fibers. Myf5 and MyoD expressions were increased in faster and slower muscles, respectively, indicating a different role of each muscle regulatory factor. On the other hand, calcineurin signaling plays an important role in the hypertrophy response through decreases in MSTN expression [40]. 

## 3. Consequences of the Inactivation of Myostatin on Conformation and Carcass Quality

**Figure 1:**
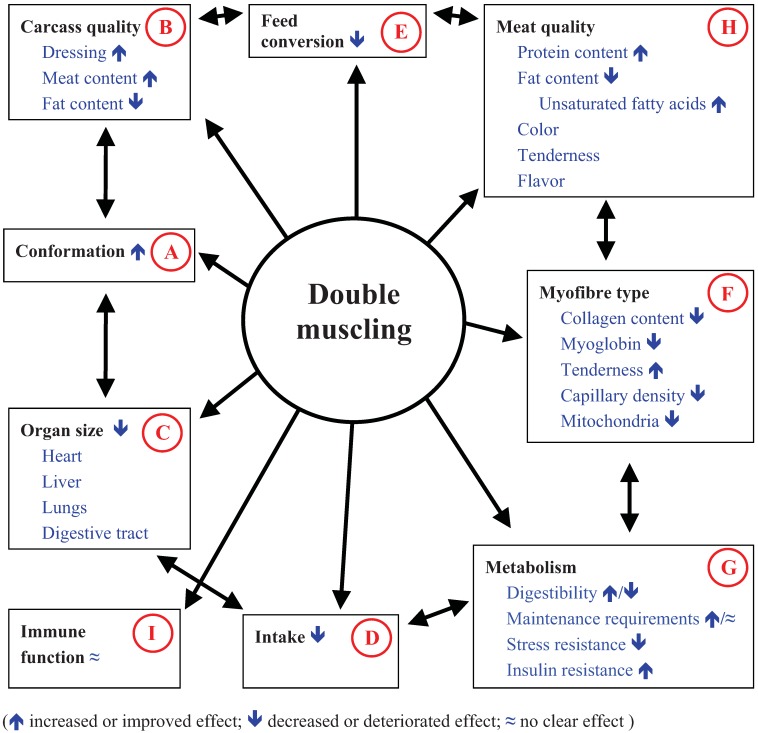
Impact of double muscling on some important animal characteristics.

The repercussions of double muscling in cattle husbandry will be elaborated in this paper. Figure 1 shows the impact of double muscling on some important animal characteristics. Several living animal characteristics and post-mortem issues are affected by MSTN. Interactions can occur, so that the mechanisms of the MSTN inactivation act in a coordinated manner.

### 3.1. Impact of Double Muscling on Conformation and Carcass Quality

An improved animal conformation means increased meat to other tissue ratio. DM animals are characterized by a better conformation (Figure 1(A)) and a hypermuscularity. These properties result in more compact animals. In fetuses of similar crown-rump lengths, double muscling increases body weight and the weight of individual muscles [41]. At carcass level, blockiness is significantly higher for DM animals [42]. Muscle hypertrophy is not similar for all muscles [43]. The variation ranges from 8 to 51% in the forequarters and from 9 to 34% in the hindquarters [44]. This hypermuscularity also occurs at the expense of bones. The limb bones are undersized in DM animals. Decrease in bone weight between DM and non-DM animals varies from 2.9 to 9.6%. Muscle hypertrophy and reduced bone weight result in an improved carcass quality (Figure 1(B)). Dressing percentage is increased in comparison with non-DM animals, concomitant with a higher lean meat content in the carcass and a lower content of fat and bone [45,46]. This means that carcass grading is different between DM and non-DM animals. Carcasses from DM animals are mostly classified as superior and excellent [46], according to the European beef carcass classification scale [47]. 

DM animals not only have a higher carcass yield, they also have a higher cutability or more expensive cuts in the meat yield. This can be demonstrated by comparing Belgian Blue bulls with a DM or a normal genotype, weighing 600 kg at slaughter. Dressing averages 70 and 64%, respectively, resulting in a cold carcass weight of 420 and 384 kg, respectively. Owing to the higher lean meat content in the carcass (76 *vs.* 65%), total meat amounts to 319 and 250 kg, respectively, being 28% more for DM animals. From this total meat yield, 171 and 123 kg are higher-priced roasts and steaks, while 148 and 127 kg are lower-priced cuts for stewing or boiling, or for hamburgers, being 39 and 17% more for DM animals within each class of retail cuts, respectively. More information about the effect of double muscling on carcass characteristics is reported by Arthur [13].

### 3.2. Impact of Double Muscling on Organ Size

Several internal organs have a reduced size in DM animals compared to non-DM animals (Figure 1(C)). Charolais DM bulls, slaughtered at 15 or 20 months, were characterized by lower weights (% reductions) for spleen (±30%), liver (±20%), heart (±20%), blood (14 and 20%, respectively) and lungs (10 and 24%, respectively), compared to non-DM bulls [48]. Similar results were found for BBDM bulls [44], with reductions varying from 10% for the adrenal glands to 15% for the heart, 17% for the liver and the digestive tract, 19% for the lungs, 37% for the spleen and 51% for the thymus. Vermorel *et al*. [49] expressed organ size as g per kg empty body weight and found a reduction of 18% for the digestive tract, 16% for the liver and the kidneys and 14% for the heart. The weights of several organs (e.g., liver, kidney, heart and digestive tract) were also significantly reduced in mice, by 12–20%, when a MSTN mutation was introduced [50]. The lower liver weight in MSTN null mice was explained by the negative feedback of hepatic-produced IGF-I on circulating GH, which in turn regulates liver size [33]. The reduced digestive tract in DM animals is clearly demonstrated by a higher ratio of empty body weight to body weight. This ratio amounted to 90 and 93%, respectively in BBDM cows [51] and bulls [52]. Ratios of 72% [53], 79–86% [54] and 84% [55] have been reported for non-DM animals. 

Besides internal organs, skin weight is also reduced in DM animals, ranging from ±20% [44,48] to 29% [49]. The reduced weight of internal organs, skin, head and legs results in a large reduction of the fifth quarter and yields a high dressing percentage, as mentioned above. In a comparison involving 10 beef cattle breeds, Asturiana de los Valles yielded the best carcass quality due to the DM condition [56]. 

The reduction in lung weight not only affects the weight of the fifth quarter and carcass weight, but it may also induce a negative effect on respiratory functions in DM animals. Bovine lungs are characterized by about 25% of lung volume per unit of body weight as compared to the mammalian mean [57]. Comparing horses with steers, horses have nearly two times larger lung volumes, a 100% larger cardiac output, and a 40% higher hemoglobin concentration [58]. The small airway resistance is significantly higher in DM calves than non-DM, which may explain the sensitivity of this cattle type to severe bronchopneumonia [59]. Furthermore, DM cattle appear to be predisposed to develop alveolar hypoxia and hypoxemia in comparison with conventional cattle [60,61]. The quantity of β-adrenoceptors in the tracheal smooth muscle membranes is lower in DM calves (*P* < 0.05) than in non-DM calves, while the density of muscarinic receptors is about 40% higher (*P* < 0.01). Inflammation of the respiratory tract in DM calves may be complicated by an imbalance between the cholinergic bronchoconstrictor and the β-adrenergic bronchodilator components of the autonomic nervous system [62], which may help to explain their sensitivity to respiratory diseases. The production of viscous purulent exudate in response to respiratory diseases often exceeds the removal by expectoration in cattle, so that the airways are obstructed and gaseous exchange is impaired [63]. Consequently, DM animals seem handicapped when there is a risk of respiratory diseases. Therefore, extra attention should be paid to the housing of DM animals. DM calves housed in outdoors hutches are mostly associated with a decreased risk of pneumonia compared to those in indoor confinement [64]. 

Tyler and Ramsey [65] found that immunoglobulin G absorption during the first 18 h was slower in hypoxic than in normoxic newborn calves. However, absorption rate increased dramatically after 18 h, and immunoglobulin G levels did not diminish until 42–48 h. The absorptive capacity was unaffected by hypoxia. Extension of the absorptive period without affecting the absorptive capacity was accomplished by the diminished rate of absorption during hypoxia. Therefore, it is assumed that immunoglobulin absorption in DM calves is not negatively affected due to their higher sensitivity to hypoxia.

The aforementioned smaller heart [44,48,49] acts in concert with the smaller lungs. Cardiac performance, capability, and reserve are reduced in DM animals compared to non-DM ones [66]. The lower cardiac performance, together with the predisposition to develop alveolar hypoxia and hypoxemia, is critical for the oxygen transport in DM cattle. Furthermore, endurance of DM cattle is lower, leading more quickly to exhaustion after severe exercise [67], which may even terminate in sudden death [68]. This phenomenon is similar to results found in peroxisome proliferator-activated receptor γ coactivator 1α (PGC-1α) knock-out mice. These mice exhibit a shift from oxidative Type I and IIA toward fast glycolytic fibers. They have a reduced endurance capacity and exhibit fiber damage and elevated markers of inflammation following treadmill running. These animals also have a reduction in mitochondrial gene expression [69]. Mice with a deletion of the PGC-1α gene may be similar to DM animals, because of an increased number of fast-twitch muscle fibers and a reduced number of mitochondria (see further). Subjecting these mice to the task of running to exhaustion, using a treadmill, resulted in a significantly lower fatigue resistance index than in wild-type mice. Furthermore, the hearts of the PGC-1α null mice were unable to mount an appropriate chronotropic response to exercise and other physiologic stimuli that activate β-adrenergic input to the heart [70]. They also traveled a significantly shorter distance. This behavior is in accordance with the reduced locomotor activity of DM animals (see further). This exhaustion is explained by a lower blood hematocrit count [67] and hemoglobin level [71], although Gustin *et al*. [72] doubted whether the greater sensitivity of DM cattle to exercise could be explained by hematological factors. Nevertheless, the robustness of DM cattle seems to be reduced, so that extra precautions are necessary with regard to animal welfare.

A consequence of the smaller digestive tract is reduced feed intake capacity (Figure 1(D)), as reported by several authors [6,73,74]. It is possible that a reduced feed intake capacity is also partly responsible for the reduced liver weight in DM animals, besides the growth hormone axis [33]. A reduced liver weight in DM animals is in line with the significantly decreased liver mass observed in steers after a nutrient deprivation [75]. When concentrate intake was reduced to about 55% of the *ad lib* intake, liver weight was decreased by 25% after a 90 kg live-weight loss. However, the relationship between reduced intake level and liver weight is not clear. An increased nutrient density of the diet is necessary for DM animals to counterbalance the reduced feed intake capacity. Furthermore, a large dietary protein concentration is necessary for the high lean meat deposition (Figure 1(B)); albeit that dietary protein is more efficiently converted into body protein. DM animals are known to be slow “turning over”, depositing large amounts of muscle protein with a greater capacity for protein synthesis than non-DM animals [76]. It is questionable if the reduced digestive tract exerts an effect on digestibility (Figure 1(G)). Clinquart *et al*. [73] found a lower dry matter digestibility (−4.6%; *P* < 0.01) in BBDM bulls compared to Holstein bulls and a tendency for a lower digestibility (−2.7%) in BBDM than in non-DM bulls. This was not confirmed in another study [74], where a tendency for a higher dry matter digestibility (1.1%; *P* = 0.10) was found for BBDM bulls compared to Belgian Blue non-DM bulls, resulting in a higher dietary energy content. However, feed intake was significantly lower for the DM genotype. When a correction was made for a different feed intake, the energy value was similar for both genotypes. A similar dry matter digestibility for DM and non-DM cattle was also reported by Vermorel *et al*. [49]. From these results it can be concluded that feed energy evaluation is not different between DM and non-DM cattle. Specific energy and protein requirements have been deduced for BBDM animals [77] based on a similar digestibility.

The reduced feed intake capacity also means that DM animals are incapable of using low quality diets efficiently. Dry matter intake is reduced and the energy content per kg dry matter is also reduced, so that there is a double negative effect on energy supply. For these reasons high-energy diets are often fed to DM animals.

Feeding BBDM cows below their requirements resulted in a mobilization of adipose tissue, but the protein balance was also negative [78]. Mobilization of body protein does not result in an efficient use of protein and can be explained by the energy demand for urea synthesis in the liver [79]. Similar results were found in bulls when they were quantitatively restricted. A similar feed restriction was more dramatic for live-weight gain and energy efficiency in DM bulls than in non-DM animals. Feed conversion was lower for Charolais DM animals than for Charolais non-DM and Friesian bulls when fed to appetite, while the opposite result was observed when fed at 75% of *ad lib* intake [80]. When a good quality diet is freely available feed conversion is lower for DM animals (Figure 1(E)), because of their reduced fat deposition. A significant interaction between diet type (concentrate or roughage based diets) and beef type (DM *vs.* non DM animals) with regard to voluntary intake was reported [81]. An interaction between nutrition and MSTN status for growth rate and myofiber characteristics has been confirmed in heterozygote lambs with a MSTN mutation [82]. Genotype-nutrition interactions cannot be isolated from body composition [83]. When *ad lib* energy intake is greater than the requirements for protein retention capacity and associated lipid deposition, a restricted energy intake reduces lipid deposition. When energy intake is just sufficient for protein retention, a restricted energy intake reduces lipid deposition and protein retention. DM animals are an example of animals with an extreme protein deposition. These findings emphasize the importance of an appropriate diet to realize a high performance in DM finishing animals.

Besides specific protein and energy requirements for DM animals [77], it cannot be excluded that some mineral requirements may also be increased. In a study sacrificing seven Belgian Blue newborn calves, seventeen bulls and twenty-four cows with diverging parities, all with DM genotype, the phosphorus content in the empty body was determined. Increasing empty body weight had no significant effect on phosphorus concentration [84], while other findings [85,86,87] were unanimous about a decreasing phosphorus accretion. It can be concluded that DM animals probably have a higher phosphorus requirement than non-DM animals, especially at a heavier weight. Diets with low levels of calcium and high levels of phosphorus are known to be calculogenic [88]. Males are at greater risks of obstruction due to urinary calculi than females, because of smaller urethras [89] so that stones pass less easily. Apart from the reduced weight of the kidneys in DM animals [44], Ouhayoun and Arnal [90] found that the medulla and the cortex were reduced as well. Furthermore, the number of urinary tubes and the diameter of the Bowman’s capsules were also reduced. This may reduce plasma ultrafiltration, so that DM bulls are more predisposed to urolithiasis. 

There is also need for an increased dietary selenium level. Guyot *et al*. [91] reported that 0.5 ppm dietary selenium may be advised for BBDM cattle to optimize health and performance, while 0.1 ppm is recommended [92]. The DM phenomenon may result in an increased susceptibility to white muscle disease or muscular dystrophy [93], explaining the higher need for selenium. 

Plasma creatine phosphokinase is a good indicator during the active stage of myodegeneration when the myofibers are disintegrating [94]. Plasma creatine phosphokinase in DM animals parallels that of non-DM animals for the first 18 hours of fasting, but continues to increase steadily throughout the fast, so that the difference between genotypes is significant by 30 hours [68]. A higher creatine phosphokinase activity in DM animals has also been reported [42,68], although the difference was not always significant [95]. These findings may confirm the predisposition of DM cattle for muscle degeneration.

### 3.3. Consequences of Double Muscling for Locomotion

According to Wolff's law, bones will adapt to their loads. Increased loads on a particular bone will result in a remodeling, so that the bone will become stronger to resist the extra load. The aforementioned smaller bone content in DM animals may have consequences for their legs. As more body weight should be carried by the reduced skeleton [44,48], bones are more highly loaded. Hendricks *et al*. [96] reported a smaller metacarpal length in 13–15 month old Angus DM bulls than in non-DM animals. Cortices tended to be thinner. Maximum shearing stress and modulus of elasticity were 15 and 18% lower, respectively, in DM than in non-DM bulls, but due to a large variation and a low number of animals, differences were not significant. The length of *humerus* and *femur* in DM Charolais bulls were significantly reduced by 4 to 5%, while femur circumference was reduced by 7 to 10% [48]. Shahin *et al*. [97] dissected DM and non-DM cows at slaughter. They found that DM cows have a lower proportion of bone in the carcass, in the entire forelimb as well as in the entire hind limb. *Humerus*, *carpus* and *ox coxa* percentages of total bone weight were significantly lower in DM cows. Moreover, work in mice showed a decrease in force generation of muscles from MSTN null mutants [98,99,100]. These MSTN null mutant mice were also characterized by increased degenerative changes in the intervertebral disc [101]. Mendias *et al*. [98] investigated the role of MSTN on the properties of tendons. Although the mass of the *tibialis anterior* and *soleus* muscles of MSTN null mice were 72 and 82% greater, respectively, than those of wild-type mice, tendons were 40 and 44% smaller, respectively, resulting in a 64 and 69% decrease in the tendon/muscle mass. The cross-sectional area of the *tibialis anterior* tendons was 50% smaller. MSTN null mice also showed a 14-fold-greater stiffness. The stiffness of tendons is a critical factor in determining the damage to muscle fibers during lengthening contractions. Consequently, the loss of MSTN had a major impact on the mechanical properties of the tendons.

Bone development is a fine-tuned process. The cells of maturing cartilage enlarge at the expense of the surrounding matrix, and the matrix itself becomes mineralized by the deposition of calcium phosphate as hydroxyapatite crystals. However, the main components of the matrix are Type I collagen fibers. The hardness of bone originates from the calcified matrix, while the strength comes from the embedded collagen fibers. Dying chondrocytes leave large empty cavities, which are invaded by osteoclasts and blood vessels, while osteoblasts deposit bone matrix. Chondrogenic differentiation and cell death involves a signaling pathway mediated by BMP-2, BMP-4 and BMP-7 [102]. Blood vessels are necessary for the transport of the nutrients for the survival of the bone. The ossification of the epiphysis may be retarded in the absence of cartilage canals [103]. Merino *et al*. [104] provided evidence for a stage-dependent specificity of the chondrogenic function of activins in the course of limb development. Activin βA and βB subunits and activin type IIA and IIB receptors exhibit a regulated pattern of expression in the different steps of joint formation, indicative of the involvement in digit arthrogenesis. We already mentioned that MSTN signals through a transforming growth factor-β/activin/nodal-like pathway. MSTN potently antagonizes BMP7, which is involved in chondrogenic differentiation and cell death. BMP receptor signaling is required for early development and creation of multiple tissues, but also for ongoing maintenance of articular cartilage after birth [105].

Rebbapragada *et al*. [27] speculated that BMP-dependent patterning of bone and cartilage within the limbs might be refined by MSTN. It may become clear that deleted MSTN has an adverse effect on cartilage and bone formation. The cartilage content in the 10th rib cut is reduced in DM animals [106].

The hooves of cattle are important for locomotion. The corium is part of the hoof containing blood vessels and nerves. At the heel, a pad of fatty tissue forms the digital cushion, playing an important role in blood circulation. It draws blood out of the foot into circulation as the heel makes contact with the ground when the animal moves. Therefore, exercise is important for new hoof horn formation, as it maintains an optimal blood circulation through the foot.

DM animals are deviant from non-DM animals, as their tissues contain less collagen. Genes encoding Type I and III collagen are down-regulated in DM muscles [107]. Consequently, bone strength may be reduced. Furthermore, capillary density is lower in DM animals. This may be critical for the nutrient support in bones and hooves. If the increased phosphorus requirement of heavier DM-animals is not fulfilled, it may be detrimental for bone formation. Therefore, precautions with regard to nutrition and confinement of DM cattle are necessary. It is remarkable that the energy cost of standing is 25% higher (*P* = 0.01) in BBDM than in non-DM bulls [49]. As a consequence, time spent standing is 13% lower in DM bulls. This may be detrimental for the blood circulation in the hooves and the formation of new horn. This is confirmed by the undersized limb bones in DM-animals [44] and the smaller circumference of the canon bone [108] and the *femur* [48]. Inhibition of collagen synthesis in connective tissues may also attenuate mechanical and structural adaptation of tendons and ligaments. 

Double muscling may be a predisposing factor to arthrogryposis [109]. Belgian Blue and Charolais breeds have a high incidence of arthrogryposis. It is a hereditary disorder caused by mutation. Genetic testing for this disorder, and six other congenital defects (congenital muscular dystonia I and II, crooked tail syndrome, dwarfism, hamartoma, and prolonged gestation) is frequently conducted in BBDM cattle to eliminate these defects, so that important economic losses can be reduced. In breeding bulls it is advised to test the presence of these mutations. 

In pens with concrete slatted floors compared to other bedding types, both the percentage of animals with hoof lesions and the severity of the lesions are increased [110]. In beef cattle, movement may be reduced on slippery floors. Movement difficulties can cause irregular hoof wearing and lameness [111]. It looks like DM cattle are more susceptible to leg injuries. In a beef production experiment with Piedmontese DM bulls housed in fully slatted-floor pens, half of the animals showed signs of lameness in the course of the finishing period [112].

As mentioned already, DM animals often receive high-energy diets with a low amount of fibrous components. These diets may provoke subacute ruminal acidosis with lameness as a consequence. Therefore, fibrousness of the diet is important for finishing DM animals fed high-energy diets. A minimum structural value of 0.6/kg dietary dry matter for BBDM beef bulls is desirable [113].

### 3.4. Impact of Double Muscling on Reproduction Performance

The previously mentioned reduction of the skeleton in DM animals, and especially the reduced development of the hip bone [48] may have repercussions on reproduction performance. Short *et al*. [114] reported a linearly decreased pelvic area (*P* < 0.05) when the MSTN allele was included in Piedmontese cattle (*P* < 0.05). Pelvic opening of DM dams was 10 and 6% lower (*P* < 0.05) than in non-DM Charolais [115] or crossbred cows [116], respectively, so that the incidence of dystocia and perinatal mortality was higher in DM cows [116]. The heavier fetal weight of BBDM cattle compared to non-DM cattle [117], rising up to 50 kg in BBDM calves at birth [118] may increase dystocia. The ratio of calf birth weight to dam weight averaged 9.0% for primiparous BBDM cows [119] *vs.* 8.3% (*P* < 0.001) for multiparous BBDM cows [120], compared to 7.5 and 6.5% for primiparous and multiparous Holstein cows, respectively [121]. The discrepancy between the underdeveloped maternal reproductive tract and the increased calf birth weight has made caesarean section an elective operation [122]. Calves born after an early surgical removal have a good vitality, blood pH and lactatemia at birth [123] close to those of eutocial calves, without negative effect on colostral immunoglobulin absorption compared to delayed surgical removal (delayed parturition after severe calf pulling). A drawback of the application of caesarean section is the significant reduction in subsequent pregnancy rate [124] resulting in an enlarged calving interval. A field study showed that the calving interval was significantly longer for BBDM cows, averaging 435 days compared to 393 days for dairy cows [125]. Moreover, each of the first three calving intervals investigated was longer for DM cows. This is longer than the average reported in the literature: three-hundred and seventy days in Angus cows [126], 365–368 days in Angus-Hereford and Charolais-Maine Anjou crossbred cows [127] and 377 days for Simmental cows, 387 days for Salers cows, 381 days Limousin cows and 393 days for Hereford cows [128]. Reduced fertility after caesarean operations may occur as a consequence of an increased incidence of uterine adhesions that affect the ovary or uterine tube and hinder involution [129]. In addition, the scar tissue formation within the uterine wall may increase the risk of abortion during subsequent pregnancies, limiting expansion of the uterus and/or hindering nutrition of the fetus. DM cows are also less able to carry fetus to term: implantation is difficult, leading to an increased embryonic mortality rate [130]. 

A longer calving interval cannot solely be attributed to caesarean section. The semen quality is significantly poorer for BBDM than for Holstein bulls [131]. Nevertheless, egg quality was better in BBDM cows than in Holstein cows [132]. However, although not significantly different, the number of transferable embryos per embryo recovery session was reduced by 31%, from 6.1 in Holstein cows to 4.2 in BBDM cows, while the number of unfertilized oocytes per embryo recovery session was increased by 39%, from 2.8 to 3.9. These results are in accordance with those obtained with mice, showing a reduced litter number in MSTN immunized mice compared to control mice, while embryo development was normal in both groups [133]. This was explained by a significantly lower number of developing follicles in ovaries of MSTN immunized mice. According to *in vitro *results of Ciarmela *et al*. [134] MSTN reduces the total number of pregnant human myometrial cells in a time- and dose-dependent manner, suggesting some functions in myometrial smooth muscle cells. Their findings also demonstrate that MSTN is expressed at higher levels only in some phases of the reproductive cycle. The pattern of MSTN expression during the estrous cycle may be regulated by steroid hormones.

From experiments with golden hamsters, a possible role of MSTN in placental development and function cannot be excluded [135]. Active MSTN may immunomodulate the uterine microenvironment. MSTN gene expression level declined as pregnancy progressed, to reach its nadir during late pregnancy. The amount of active MSTN peptide also declined, suggesting that MSTN is more active in early pregnancy, the phase with the implantation of the embryo. Therefore, the release of MSTN into the uterine environment is beneficial to the developing conceptus. It is possible that the inactivation of MSTN in DM cattle may compromise fetal development. 

Activins influence several physiological processes in adults, among others reproduction in the male and the female [136,137]. Muttukrishna *et al*. [138] described the pituitary ovarian axis, where activin A has an endocrine and activin B has an autocrine/paracrine role on regulating pituitary FSH secretion. In the case of early pregnancy loss they referred to significantly lower levels of activin A when the miscarriage was complete. Maternally derived activin subunits, predominantly βA, are specifically up-regulated in the uterine luminal epithelium during pre-implantation embryo development, and subsequently in the endometrium, during the critical development events of gastrulation and organogenesis [139]. It is suggested that activins secreted by the oviduct and uterus could facilitate the development of the embryo prior to implantation. Because MSTN and activins are structurally related, it should not be amazing that MSTN may affect bovine fertility and reproduction.

Heat stress can negatively affect aspects of reproduction [140]. DM cattle are more susceptible to heat stress than non-DM cattle. Rectal temperature of cattle increases with rising ambient temperature, but the difference between DM and non-DM animals becomes larger with increasing ambient temperature [141]. The lower capacity of DM animals for heat dissipation is due to their blockiness and the smaller body exchange surface per body mass unit. Body heat is generated in proportion to muscle mass or volume, while body heat is dissipated in proportion to body surface area. Furthermore, DM cattle are characterized by a lower capillary density [142]. Blood flow to the skin is positively correlated to the sweating rate [143], and sweating is an important thermoregulatory mechanism to dissipate excess body heat. As the ease with which metabolic heat is moved to the skin for dissipation depends on the amount of heat traveling in the blood from the body core to the skin, the reduced capillary density is a disadvantage for DM animals. Reynolds *et al*. [144] reported the effect of heat stress in pregnant cows. On day 100 of gestation non-DM Hereford cows were confined in a heated barn at 16 °C or in individual stalls with a diurnal temperature regimen of 28 °C for 12 h and 36 °C for 12 h and a dewpoint of 21 °C. Uterine and umbilical blood flows were estimated on day 169. They were reduced by 34 and 23%, respectively, in heat stressed cows, and fetal weights amounted to 82% of those of non-stressed cows. In any case, capillarity is reduced in DM cattle. Additional heat stress may further reduce blood flow in these animals, which may increase the detrimental effect on fetal development.

Besides over the body surface, heat loss can also occur via the lungs. About 15% of the endogenous heat is lost directly from the body core via the respiratory tract in cattle [145]. Consequently, the reduced lung capacity, as mentioned before, is another disadvantage for DM animals. Furthermore, cattle that had severe respiratory disease early in life will have a decreased ability to regulate their heat load. Because DM cattle are more sensitive to severe bronchopneumonia [59], relatively more DM animals will have to contend with heat stress.

### 3.5. Influence of Double Muscling on Histological Aspects of Skeletal Muscle

Growth and development is a complex process involving the cell cycle regulation with progression through the G1, S, G2 and M phases [146]. Muscle fibers are issued from myoblasts which proliferate, then fuse to form myotubes, and finally differentiate into muscle fibers. At least two generations of cells are involved in bovine myogenesis. These cells are different by the expression of the different myosin heavy chain isoforms and by their size. Cells from the primary and secondary generations proliferate until 180 days after conception. The total fiber number is fixed from this stage on. Therefore, the fetal stage is crucial for skeletal muscle development. The first generation of myoblasts is completely differentiated from the end of the second trimester. The second generation achieves its differentiation at the end of fetal life. The first generation of myoblasts will give rise to Type I fibers after birth, while the second generation of myoblasts will be converted mainly into fast fibers, but also slow fibers depending on the muscle type [147]. The cell cycle progression is affected by biologically active MSTN, with an arrest in the G1 and G2 phases. Consequently, cells are not allowed to make the transition to the S phase where DNA synthesis occurs, and the M phase where cell division occurs, respectively. At a cellular level, MSTN affects cell cycling by controlling the entry of satellite cells into the S phase [148,149,150]. Intact MSTN is thus a regulator in the development of fast glycolytic myofibers [151]. MSTN expression increases during myoblast differentiation [38]. 

In DM animals with inactive MSTN, muscle fiber number [19,41,96,142,152,153] and the number of fast glycolytic fibers were significantly increased [142,153,154,155,156]; the same was found in MSTN knockout mice [157], emphasizing an extensive hyperplasia. This can be explained by the lack of inhibition of myoblast proliferation and differentiation by MSTN. MSTN expression was detected in bovine embryos from day 15 of pregnancy and increased from day 31 onwards [158] and is regulated throughout pregnancy [159]. At 100 days after conception, BBDM fetuses already present muscle hypertrophy, which originates from a higher myoblast proliferation [160]. Accordingly, MSTN expression was found in the latest differentiating cells from the second generation [159]. Furthermore, the *semitendinosus* muscle contained a smaller proportion of primary fibers and a higher proportion of secondary fibers, resulting principally in fast fibers in adult muscle. This greater proliferation is accompanied by a delay in the differentiation phase [147,161]. Picard *et al*. [162] found that differentiation of muscle fibers was slower in DM fetuses, particularly during the first two-thirds of gestation. DM animals showed a slower rate of differentiation at 90 days after conception in first generation cells, while differentiation was mainly retarded in second generation cells at 130 days after conception. Differentiation of first generation cells was no longer different between DM and non-DM animals. Cells of the *longissimus* muscle of DM fetuses displayed a greater proliferating capacity and faster proliferating and alignment phases, but this advance is apparently lost after alignment, and the fusion of the DM myoblasts is delayed [163]. As a consequence, muscles of adult DM animals exhibit a higher proportion of fast glycolytic fibers, simultaneously with a higher total number of fibers compared to muscles of non-DM cattle breeds [151] (Figure 1(F)). The overall increase in muscle mass, which is due to an increase in the number of muscle fibers (hyperplasia) and to a lesser extent to fiber enlargement (hypertrophy), differs between muscles. Muscle fibers tended to be larger in the *rectus abdominis* muscle, while fibers in the *semitendinsus* muscle, especially Type I fibers, of DM animals were smaller [155]. The myofiber cross-sectional area within the fiber type was smaller in DM than in non-DM bulls. Due to the higher occurrence of fast glycolytic fibers, mean fiber size is larger in DM animals. The modified histological aspects may have some repercussions. Muscles with fast glycolytic fibers are characterized by an increased fiber sectional area and glycogen content, and a reduction of the content of myoglobin, fat, collagen, the number of mitochondria, and the capillary density [164]. Myoglobin is one of the first identified gene targets of calcineurin, and is expressed in highly-oxidative Type IIA fibers [40]. Calcineurin activation is sufficient to induce the slow fiber gene regulatory program *in vivo*, but additional signals may be required for skeletal muscle hypertrophy [165]. Transcriptional co-activator peroxisome proliferator-activated receptor γ coactivator 1α (PGC-1α) is a principal factor regulating muscle fiber type determination in skeletal muscle. PGC-1α is expressed preferentially in muscle enriched in Type I fibers [166].

### 3.6. Influence of Double Muscling on Metabolism

The aforementioned shift towards faster glycolytic myofibers in DM animals (Figure 1(F)) is responsible for a shift from an oxidative to a more glycolytic metabolism [164]. This coincided with significantly less aerobic and more anaerobic fiber ratios in DM compared to non-DM Belgian Blue bulls [155]. The anaerobic or glycolytic metabolism is in agreement with the reduced number of mitochondria in fast glycolytic myofibers. Considerable evidence shows that PGC-1α is sufficient to promote mitochondrial biogenesis and regulate mitochondrial respiratory capacity [70]. The increased number of fast-twitch muscle fibers in DM animals and the relationship between this myofiber type and PGC-1α [166] may lead to fewer mitochondria. It is noted that the lower number of mitochondria is not due a lower expression of the mitochondria themselves, but to the glycolytic muscle type containing less mitochondria [167]. Energy is generated in the mitochondria from the breakdown of glucose or fatty acids and the conversion to ATP in the Krebs cycle. Oxygen is of prime importance for muscle energy metabolism in the mitochondria. Factors involved in meeting the oxygen requirements are blood flow, arterial oxygen concentrations, tissue extraction rates, myoglobin content and mitochondrial volume [168]. After activation in the cytoplasm fatty acids are oxidized in the mitochondria through a cycle of different reactions mediated by enzymes. However, as the number of mitochondria is reduced in DM animals, there is less opportunity for fatty acid oxidation. This is in accordance with the low fat content in DM animals, so that the amount of fatty acids that should be oxidized after breakdown is very limited. Furthermore, due to the reduction of the capillary density, the oxygen supply is also reduced and the elimination rate of lactate may be impaired. The hemoglobin in the red blood cells and myoglobin inside muscle cells act in tandem for oxygen transport. However, myoglobin content is lower in DM than in non-DM finishing cattle [46,155]. The hemoglobin content in blood was not different between DM and non-DM dairy animals weighing between 40 and 680 kg [72], while the hemoglobin content tended to be lower in DM calves compared to Friesian neonatal calves, and the difference was even significant in calves between 2 and 28 days of age [61]. As a consequence, lactate can accumulate, especially after exercise [169]. Even in rest, blood lactate concentrations are significantly higher in DM animals, although this was not always confirmed [46]. 

Fast twitch glycolytic muscles have a much lower protein turnover than slow-twitch oxidative muscles with more Type I fibers [170]. Based on the indirect calorimetry technique to measure metabolic rate in MSTN null mice, total and resting oxygen consumption were 14% and 8% higher, respectively, compared with wild-type mice, but their body weight was also higher [171]. However, MSTN null mice had lower (*P* < 0.01) rates of total and resting oxygen consumption if results were expressed as a function of body weight. Results of Guo *et al*. [172] confirmed that MSTN null mice have a lower metabolic rate when normalized to total body weight. Tu *et al*. [173] used double knockout mice and also found a lower metabolic rate compared with controls, if oxygen consumption was expressed as a function of lean mass weight. This means that maintenance requirements and feed intake capacity may both be modified in the same direction (Figure 1(G)). 

Visceral organs are considerably smaller in DM than in non-DM animals, as previously reported (Figure 1(C)). Ferrell [174] reported that the mass of these organs was highly correlated with energy expenditure. Even though visceral organs constitute less than 10% of body mass, they account for 40 to 50% of total energy expenditure. Therefore, it can be assumed that DM animals have a lower energy expenditure than non-DM animals. However, the daily energy expenditure of BBDM bulls was similar to that of non-DM bulls [49]. These results are not in agreement with previous findings [175], based on indirect calorimetry measurement in DM and phenotypically normal bulls. The metabolic rate was significantly higher in DM bulls and found to be 415.0 *vs.* 365.9 kJ/kg body weight daily. Moreover, this is not in accordance with the slower turnover rate of fast glycolytic myofibers, the lower feed intake capacity, and the reduced liver size of DM animals.

MSTN null mice have an improved glucose metabolism and an increased insulin sensitivity than non-transgenic control mice [173]. A higher insulin sensitivity, which was also reported for BBDM bulls [176], may be responsible for a reduced adipogenesis. BBDM calves have lower basal glucose concentrations and insulin peak concentrations, and higher glucose elimination rates than Holstein-Friesian calves [177]. MSTN up-regulates several genes involved in the regulation of the glucose metabolism [178]. The improved insulin sensitivity of DM animals is due to the AMP activated protein kinase for the promotion of glycolysis, as shown in C2C12 mouse skeletal muscle cells and L6 rat skeletal muscle cells. MSTN specifically inhibits glucose uptake into BeWo cells, a placental cell line, suggesting that MSTN may control glucose metabolism within the placenta [179]. This inhibition may be withdrawn in case of inactive MSTN. Significantly lower basal glucose and insulin levels after an overnight fast were reported for BBDM calves (4.3 ± 0.67 mmol/L and 5.1 ± 3.56 mU/mL, respectively), compared to 5.9 ± 0.86 mmol/L and 8.7 ± 5.81 mU/ml for Holstein calves [180]. After infusion of 150 mg glucose per kg body weight, a comparable increase in glucose was seen for BBDM (1.8 ± 0.63 mmol/L) and HF calves (1.6 ± 0.56 mmol/L). The increase of insulin was significantly higher in HF (27.6 ± 17.43 mU/mL) than in BBDM (18.9 ± 18.9 mU/mL) calves. As a result, the insulinogenic index, as a measure of early insulin secretion, was significantly higher in Holstein than in BBDM calves. The higher insulinogenic index of Holstein calves may be an indication of a lower insulin sensitivity, as HF calves have to produce more insulin to maintain normal glucose levels. Lower serum insulin levels were also reported for DM Piedmontese bulls in comparison with non-DM Piedmontese and Friesian bulls [181]. Istasse *et al*. [182] found a tendency for a higher plasma insulin level in BBDM bulls than in Holstein bulls at a younger age, but the opposite was found in older animals. Muscle fiber Type I coincides with a higher level of sensitivity to insulin since the increase in mitochondrial protein expression is demonstrated in response to insulin. In accordance, skeletal muscle glucose uptake is regulated through insulin-dependent regulation of mitochondrial gene expression. Insulin stimulates the biogenesis of mitochondria in muscle cells [183]. As mentioned earlier, PGC-1α drives the formation of slow-twitch muscle fibers in skeletal muscle [164]. PGC-1α null mice were less susceptible to diet-induced insulin resistance [70]. This phenomenon corresponds with the reduced insulin resistance in DM cattle.

### 3.7. Influence of Double Muscling on Stress Susceptibility

DM cattle have more problems with peaks of physical stress than non-DM cattle (Figure 1(G)). As mentioned previously, forced exercise leads to exhaustion more rapidly [67,68]. These findings were confirmed by Ploquin *et al*. [184], where an endurance test resulted in differential effects on redox homeostasis and mitochondrial function. The muscle fiber type and the lower capillary density lead to an accumulation of lactic acid. Another consequence of the reduced capillary density is the difficulty for DM animals to eliminate their body heat. Heat is transported from inside to the body surface through blood vessels [145]. Furthermore, body surface per unit of body weight is also lower in DM animals, so that they are more susceptible to heat stress [141]. The reduced body area combined with a lower capillary density will decrease evaporative cooling.

As DM animals are characterized by a reduced body fat content, we may expect a more severe effect of cold stress. Homeothermic animals cope with cold environmental temperatures by their ability to make the necessary physiological and metabolic adaptations. They may increase heat production, that is fuelled by an enhanced feed intake and/or mobilize their body reserves. Protein turnover may also contribute to cold-induced thermogenesis. As mentioned above in case of DM animals, feed intake capacity is reduced. The ability for tissue mobilization is clearly reduced [78]. Furthermore, their increased content of fast glycolytic myofibers, suggests a lower protein turnover [170]. Lammoglia *et al*. [185] compared the effect of a cold environment in newborn Piedmontese crossbred calves that contained at least one copy of the Piedmontese MSTN allele with non-DM Hereford crossbred calves. At 5 h of age, calves were placed in a 0 °C room for 140 minutes, and rectal temperatures and blood samples were obtained at 10- and 20-minute intervals. Breed × time interactions were observed for rectal temperature and serum concentrations of cortisol and glucose in newborn calves. Differences in rectal temperatures and blood concentrations of glucose and cortisol suggest possible differences in thermogenic mechanisms between calves with muscular hypertrophy and normal-muscled calves. Mice with a deletion of the PGC-1α gene exhibited a marked drop in core body temperature compared to the wild-type controls when subjected to cold exposure (4 °C) for 5 h [70]. The deletion of the PGC-1α gene, resulting in more fast-twitch muscle fibers, may show a lot of similarities with DM animals. However, body temperatures of MSTN null mice and wild-type mice either under normal conditions or in response to a 60-minute cold tolerance test were similar [171].

The effect of nutritional stress was studied in DM and non-DM Aberdeen Angus bulls [186]. Animals were fed *ad libitum* or received 125 or 85% of their maintenance requirement in two equal meals per day for 18 days. Blood samples were collected at 4-h intervals during the next 2 days. Glucose concentrations tended (*P* < 0.10) to be different between genotypes at 85% intake of their maintenance requirement. Non-esterified fatty acid fluctuations in DM animals responded less rapidly after the meals, suggesting a lower ability to mobilize fatty acids in response to short term alterations in energy availability. The weak response of non-esterified fatty acids after energy restriction was confirmed with BBDM cows. Energy restrictions up to 30% of the requirements during a 140-day indoor period did not significantly affect blood concentration of non-esterified fatty acids at the end of the restriction period [78].

MSTN inactivation attenuates the changes with acute daily psychological stress [187]. Three days of daily restraint of 1 h resulted in a lower decrease in body mass in MSTN null mice than in wild-type mice, while there were no decreases in muscle mass of *tibialis anterior* and *soleus* in contradiction with wild-type mice. MSTN null mice showed a non-significant increase in serum levels of corticosterone and glucose with daily restraint, while serum levels were significantly increased in wild-type mice.

Apart from their susceptibility to lameness and respiratory disorders, there is no clear indication that DM animals have a reduced immune function. Reductions in total body fat are correlated with impaired immunity, and experimental reductions in body fat can impair antibody formation [188]. Therefore, the reduced body fat content in DM animals may suggest such an impaired immune function. Furthermore, MSTN expression was detected in spleens, suggesting that it may play an important role in modulating immune system function during conditions of stress although the precise immune function of MSTN is unknown [189]. Consequently, it can be assumed that the inactive MSTN in DM animals may exert a negative effect on the immune function (Figure 1(I)). However, there is no evidence of any deficiency in the immune response of DM calves based on the lymphocyte function when compared with non-DM calves [190].

### 3.8. Effect of Double Muscling on Meat Quality

Several authors have made allusion to the repercussion of a modified muscle fiber type on meat quality [168,191,192]; (Figure 1(F)). There are several modes of action to affect meat quality. We already mentioned the reduced myoglobin content and its impact on oxygen transport. Myoglobin also affects meat color. It is well known that DM animals yield paler meat compared to non-DM animals [46,147]. The glycolytic fibers can result in a faster decline of pH *post-mortem* [155]. Strath *et al*. [193] compared the glucose pool size in phenotypically categorized bulls as either DM animals or normal to moderate in muscling. Glucose pool size was significantly less in DM cattle, which was expressed by a higher ultimate pH [42]. A reduced glucose pool and a faster glycolysis may accelerate glucose depletion in DM animals, especially in stress situations, so that they may be slightly predisposed to produce DFD meat. An increased frequency of DFD can occur as a consequence of heat stress [194], and the increased susceptibility of DM animals for heat stress may be an additional disadvantage for DM animals. Some authors mentioned an adverse meat quality in DM animals due to a pale and two-toned color in deeper parts of the hindquarter [195,196]. A very fast grey discoloration shortly after cutting is typical for this phenomenon. At 5 h *post-mortem*, the temperature of the inner *biceps femoris* was still greater than 35 °C, while the pH was decreased to less than 5.5. Initial L* values (lightness) amounted to 36.9 and 50.1 for the outer and the inner *biceps femoris*, respectively, and were significantly different, illustrating the problem of two-toning. L* values increased during a 10 day display, but the increase was most significant for the inner *biceps femoris*, while a* values (redness) decreased. Hot boning exerted a positive effect on pH decline. At 5 h *post-mortem* the pH averaged 5.93 for the hot boned inner *biceps femoris*: compared to 5.42 for the cold boned inner *biceps femoris*. There was no period of heat toughening as a consequence of the hot boning, while a duration of heat toughening of 3.9 h was estimated for the cold boning [195]. Traditional chilling of large beef muscles is detrimental for color stability, as the rapid pH decline with higher meat temperatures in the deep muscle causes more protein denaturation, and a lower color stability and water holding capacity [197]. *Post-mortem* temperature evolution showed a tendency for higher values (*P* < 0.10) in muscles of DM animals [198]. The temperature was probably higher in DM animals because of the large muscle mass. Carcass weights of 600 kg are not exceptional for BBDM cows. The combination of a temperature greater than 35 °C and a pH of less than 6 (heat shortening), causing early exhaustion of proteolytic activity, may lead to increased toughness [199].

The higher muscle temperature in carcasses of DM animals means that the metabolism may continue at a faster rate than in non-DM animals, resulting in a lower muscle pH. Batjoens *et al*. [155] observed a more rapid pH fall *post-mortem* in DM animals. During *post-mortem* pH decline values were higher in DM carcasses compared to non-DM carcasses in experiments of Pringle *et al*. [198]. These higher pH values are in accordance with the small but significantly higher ultimate pH for DM animals [42]. Steen *et al*. [200] reported pH values of 5.54 at 3 h *post-mortem* for DM animals, while other DM animals had an ultimate pH of 5.86, so that this meat could be regarded as dry, firm and dark. The increased levels of denatured creatine phosphokinase, 34 kDa and 36 kDa components, found in SDS-PAGE patterns of the myofibrillar fraction, may be related to the higher rate of pH decline [42].

The general conclusion from most studies is that DM cattle have more tender meat than non-DM animals [13,201]. Fast glycolytic fibers imply a lower collagen content. Collagen is associated with the background meat toughness, which may partly explain the lower toughness of meat from DM animals. A lower collagen content for DM animals was confirmed by several experiments [42,202,203,204,205]. Soluble collagen content, expressed as a percentage of total collagen, was mostly not affected [204,205], although a higher collagen solubility [202] or a less mature collagen [203] was also reported for DM animals. Nevertheless, meat of DM animals was not always more tender than that of non-DM animals [42,156,206]. Within BBDM bulls, Warner-Bratzler shear force value can also differ (*P* < 0.1) due to a different livestock management [207]. The shear force value was reduced by 26% when bulls were finished after a feed restriction period of 6 months yielding a daily gain of 0.5 kg, compared to bulls fed permanently on a concentrate diet, although their body weight was similar, but they were 4.5 months older at slaughter. In any case, results from different experiments [42,200,207,208] show a large variation in shear force value of meat from DM animals. This emphasizes the need for appropriate slaughter conditions. Indeed, overall tenderness is also dependent on slaughter conditions (the rigor period) and the extent of proteolysis. Pre-slaughter stress of fasting for 48 h resulted in a significantly higher pH in DM cattle, provoking some animals with dark cutting [68]. Different calpain and calpastatin activities between BBDM and non-DM animals were reported [42]. µ-Calpain was lower in DM bulls, indicating a lower proteolytic activity, which can explain the lower meat tenderness. However, calpastatin was also lower in DM bulls, but the µ-calpain/calpastatin ratio was also lower in DM animals, suggesting a reduced efficiency of µ-calpain. Only µ-calpain activity in *longissimus* and *biceps femoris* muscles was lower in Piedmontese DM bulls compared to non-DM Angus steers [198]. Besides, cathepsin B and L concentrations in the *longissimus* muscle on 1 and 8 days *post-mortem* were also lower in BBDM bulls [42]. These results were not confirmed by Caballero *et al*. [10] who found that the activity of cathepsins B, B + L, D and H in the cytosolic compartment of the *longissimus* muscle was higher in DM bulls of the Asturiana de los Valles breed at 3 and 7 days *post-mortem* than in non-DM animals. At 14 days *post-mortem* enzyme activity tended to be lower in DM animals with significant differences for cathepsin D, meaning that the tenderisation rate was faster in DM animals. Steen *et al*. [200] found that calpain and calpastatin activity in the *longissimus* muscle varied considerably within BBDM bulls between years, although animals were finished on similar diets and slaughtered in similar conditions. Average sarcomere length amounted to 1.76 µm, ranging from 1.26 to 2.26 µm. It was similar to the length reported by Uytterhaeghen *et al*. [42]. The latter authors confirmed a larger variation of sarcomere length within DM animals. Animals were fasted for 20–24 h prior to slaughter in both experiments. Taking into account that fasting for 48 h may have a negative effect on meat quality, measured in the *triceps longus* muscle [68], it is not clear if fasting for 24 h can also exert a negative effect on meat tenderness. Sañudo *et al*. [205] reported a significant interaction between breed and slaughter weight. DM animals had the lowest shear force values at a live weight of 300–350 kg, while values were higher at 530–560 kg. Management of the finishing animals was mostly not mentioned. However, pre-slaughter growth rate of beef cattle can affect meat tenderness. A rapid growth rate results in an elevation of both muscle protein synthesis and degradation in the growing animal and an increased proteolysis *post-mortem* and an improved ultimate tenderness [199]. Furthermore, the divergence between the authors can be partly explained by the different muscles studied.

Twenty-nine and 49% of the variation in juiciness in the *longissimus* and the *semitendinosus* muscles, respectively, can be explained by the DJ-1 protein in Salers [209]. DJ-1 protein is associated with cancer and Parkinson’s disease [210]. The cytoplasmic protein DJ-1 has a protective function and changed significantly in abundance during the early *post-mortem* storage period. It acts against oxidative stress and cell death. The relationship between DJ-1 protein and meat quality traits is scarcely reported, but *post-mortem* changes of DJ-1 protein are correlated to Warner-Bratzler shear force, drip loss, and meat color [211]. Muscles with a lighter meat had increased abundance of DJ-1 protein [212], but its effect on tenderness was not clearly demonstrated [213]. Chelh *et al.* [214] showed an up-regulation of DJ-1 protein in MSTN-null mice. Consequently, juiciness may be negatively correlated with this protein. However, juiciness was not significantly different between DM and non-DM British South Devon cattle, but there was a trend for a lower juiciness in DM animals [206]. 

Meat from DM animals is characterized by a low intramuscular fat content [46,206,215,216], resulting in an intramuscular fat content of less than 1%. Besides the lower fat content, fatty acid composition is also different from intramuscular fat in non-DM animals. The concentration of polyunsaturated fatty acids (PUFA) is increased, while the concentration of saturated fatty acids (SFA) is decreased, resulting in a higher PUFA/SFA ratio [206,215,216]. This is in accordance with the fact that cell membranes are relatively more important as a fat source in DM animals. They are mainly composed of phospholipids, and phospholipids are particularly rich in PUFA’s. Furthermore, it has been shown that the metabolism of n-6 and n-3 fatty acid is affected by the MSTN gene and its effect is most evident in n-3 PUFAs of the intramuscular fat compared to intermuscular and subcutaneous fat [217]. Although the absolute content of total n-3 fatty acids is lower in meat of DM animals, they show greater deposition rates of n-3 elongation and desaturation products. This PUFA/SFA ratio together with a decreased ratio of n-6/n-3 PUFA’s [215] means that DM animals yield healthy meat. 

## 4. Conclusions

DM animals may provide some advantages for farmers, meat industry and consumers, such as an efficient conversion of feed into high valuable carcasses, a tender meat with a low fat content, indicating a meat that is healthier than normal. However, DM animals are also characterized to some extent by a lower robustness. They are more susceptible to dystocia, respiratory disease, lameness, muscle degeneration, and heat stress. Therefore, some extra precautions are needed to preserve animal welfare: appropriate nutrition to reduce nutritional stress, proper confinement to reduce respiratory disease and locomotion problems, shade during summer grazing to reduce heat stress, or elective use of caesarean section to reduce calf loss as a consequence of dystocia. Furthermore, adequate slaughter conditions are required to prevent cold shortening and heat toughening. The maturation rate of meat of DM animals is faster. Although meat of DM animals has a lower collagen content it is not always more tender than that of non-DM animals. The lower intramuscular fat content of DM animals may reduce meat flavor. It may not always be easy to find a balance between the different disciplines underlying the livestock husbandry of DM animals to obtain good performance and health, and lean meat with acceptable tenderness, flavor and juiciness.

Knowledge of the DM phenomenon has considerably increased during the last decade owing to molecular biology. It is clear that mechanisms due to the inactivation of the MSTN gene work in a coordinated manner. This means that highly qualified actors are necessary to realize good animal performance and high valuable carcasses and meat pieces. Further research may help to fine-tune the subsequent processes from birth of DM animals to the supply of healthy meat for the consumer.
